# MEK inhibition suppresses *K-Ras* wild-type cholangiocarcinoma in vitro and in vivo via inhibiting cell proliferation and modulating tumor microenvironment

**DOI:** 10.1038/s41419-019-1389-4

**Published:** 2019-02-11

**Authors:** Pan Wang, Xinhua Song, Kirsten Utpatel, Runze Shang, Yoon Mee Yang, Meng Xu, Jie Zhang, Li Che, John Gordan, Antonio Cigliano, Ekihiro Seki, Matthias Evert, Diego F. Calvisi, Xiaosong Hu, Xin Chen

**Affiliations:** 1 Beijing Advanced Innovation Center for Food Nutrition and Human Health, College of Food Science and Nutritional Engineering, China Agricultural University, Beijing, China; 2 Department of Bioengineering and Therapeutic Sciences, and Liver Center, University of California, San Francisco, CA USA; 3 Institute of Pathology, University of Regensburg, Regensburg, Germany; 4 Department of Hepatobiliary Surgery, Xijing Hospital, Air Force Military Medical University, Xi’an, China; 5 Division of Digestive and Liver Diseases, Department of Medicine, Department of Biomedical Sciences, Cedars-Sinai Medical Center, Los Angeles, CA USA; 6 Department of Hepatobiliary Surgery, The First Affiliated Hospital of Xi’an Jiaotong University, Xi’an Jiaotong University, Xi’an, China; 7 Department of Thoracic Oncology II, Key Laboratory of Carcinogenesis and Translational Research (Ministry of Education), Peking University Cancer Hospital & Institute, Beijing, China; 8 Department of Medicine, University of California, San Francisco, CA USA; 9 Institute of Pathology, University of Greifswald, Greifswald, Germany

## Abstract

PD901, a MEK inhibitor, has been demonstrated of therapeutic efficacy against cholangiocarcinoma (CCA) harboring *K-Ras* oncogenic mutations. However, most CCA exhibit no *K-Ras* mutations. In the current study, we investigated the therapeutic potential of PD901, either alone or in combination with the pan-mTOR inhibitor MLN0128, for the treatment of *K-Ras* wild-type CCA in vitro using human CCA cell lines, and in vivo using AKT/YapS127A CCA mouse model. We discovered that in vitro, PD901 treatment strongly inhibited CCA cell proliferation, and combined PD901 and MLN0128 therapy further increased growth inhibition. In vivo, treatment of PD901 alone triggered tumor regression, which was not further increased when the two drugs were administered simultaneously. Mechanistically, PD901 efficiently hampered ERK activation in vitro and in vivo, leading to strong inhibition of CCA tumor cell cycle progression. Intriguingly, we discovered that PD901, but not MLN0128 treatment resulted in changes affecting the vasculature and cancer-associated fibroblasts in AKT/YapS127A mouse lesions. It led to the decreased hypoxia within tumor lesions, which may further enhance the anti-cell proliferation activities of PD901. Altogether, our study demonstrates that MEK inhibitors could be effective for the treatment of *K-Ras* wild-type CCA via inhibiting cell proliferation and modulating tumor microenvironment.

## Introduction

Cholangiocarcinoma (CCA) is the second most common type of primary liver cancer^1,2^. Epidemiologic evidence indicates that CCA incidence and mortality rate have been increasing steadily in the past few decades^3^. CCA is a lethal malignancy, with the 5-year overall survival rate being only ~15% (www.cancer.org). Surgical resection and liver transplantation are the only effective treatment options for early-stage disease, but most CCA patients are diagnosed at advanced stages^1^. For unresectable CCA, combined administration of Gemcitabine and Platin-based drugs is the standard first line chemotherapy^4,5^. However, the response to such treatment is limited and it confers a median overall survival of only 11.7 months^1,6^. Therefore, novel and effective therapeutic strategies against CCA are urgently needed.

The Ras/Raf/MEK/ERK pathway plays a central role in regulating multiple cellular processes including proliferation, survival, and differentiation^7,8^. This pathway has been implicated as oncogenic cascade in all major tumor types, including CCA^9^. Indeed, in our previous study, we demonstrated that Ras/MAPK cascade is ubiquitously activated in human CCA with or without mutant *K-Ras*^10^. MEK is a central player of this pathway, as it transduces signals generated from extracellular molecules, including growth factors and cytokines downstream of Ras. When MEK is inhibited, it leads to repression of cell proliferation and increased apoptosis. While Ras proteins are difficult targets, MEK has become an attractive candidate to inhibit this important oncogenic pathway^11^. Currently, MEK inhibitors are intensively investigated in vitro, in preclinical models as well as tested in clinical trials^12–14^. In a recent study, we assessed the importance of MEK inhibitors, including PD901, U0126, and Selumetinib for the treatment of *K-Ras* mutant CCA. We showed that MEK inhibitors effectively reduce CCA cell growth in culture and induce apoptosis in a murine CCA model generated by the co-expression of activated mutant forms of *K-Ras* and Notch1 (KRas/NICD)^10^. Intriguingly, our study revealed that treatment with MEK inhibitors also led to decreased growth in CCA cell lines with wild-type *K-Ras* in culture^10^. Although genomic analyses showed that *K-Ras* mutations occur in ~20% of CCA^15^, sustained activation of MEK/ERK downstream effectors was detected in most CCA^10^, implying induction of this oncogenic cascade mainly in the presence of wild-type *K-Ras* in this tumor type. Consequently, it would be of high importance to determine whether MEK inhibitors are also effective in suppressing the growth of CCA with wild-type *K-Ras* alleles.

The phosphoinositide-3-kinase/protein kinase-B/mammalian target of rapamycin (PI3K/AKT/mTOR) signaling cascade is another critical intracellular pathway regulating cell proliferation, differentiation, cellular metabolism, and survival^16^. Being one of the most frequently activated signaling pathways in tumor cells, numerous efforts have been made to develop PI3K/AKT/mTOR targeted therapies^17^. MLN0128 is an ATP-competitive inhibitor, which provides a stronger blockade of mTOR signaling via suppression of both mTORC1 and mTORC2 complexes^18^. MLN0128 is currently being evaluated in several phase I and II clinical trials as a single agent or in combination therapies (https://clinicaltrials.gov/). In a previous investigation, we found that MLN0128 treatment results in a stable disease using a murine CCA model generated by activated forms of AKT and Yap (AKT/YapS127A)^19^. Mechanistically, MLN0128 efficiently inhibited AKT/mTOR signaling and induced strong CCA cell apoptosis, with limited effects on tumor cells proliferation^19^. Recent in vitro and in vivo data indicate that the PI3K/AKT/mTOR and Ras/Raf/MEK/ERK signaling pathways are interconnected through multiple points of convergence. Therefore, there is compelling evidence supporting the therapeutic strategy of dual inhibition of these pathways^20^. Tumor microenvironment has been reported to play an important role in tumor development and progression^21^. The tumor microenvironment consists of cancer associated fibroblasts and endothelial cells, which form the vasculature within the tumor nodule as well as infiltrating immune cells. Here, we hypothesized that both PI3K/mTOR and MEK/ERK pathways may function via regulating tumor microenvironment during CCA development.

In the present study, we sought to determine the therapeutic potential of a MEK inhibitor, namely PD901, either alone or in combination with the pan-mTOR inhibitor MLN0128 for the treatment of *K-Ras* wild-type CCA in vitro using human CCA cell lines, and in vivo using AKT/YapS127A CCA mice. Our study suggests that the Ras/MEK pathway is a major regulator of cell growth in CCA through both cell autonomous and cell non-autonomous mechanisms. MEK inhibitors might be effective for the treatment of *K-Ras* wild-type CCA via inhibiting cell proliferation and modulating tumor microenvironment.

## Results

### Ras/MAPK, but not AKT/mTOR pathway, is the major regulator of *K-Ras* wild-type CCA cell proliferation in vitro

**Fig. 1:**
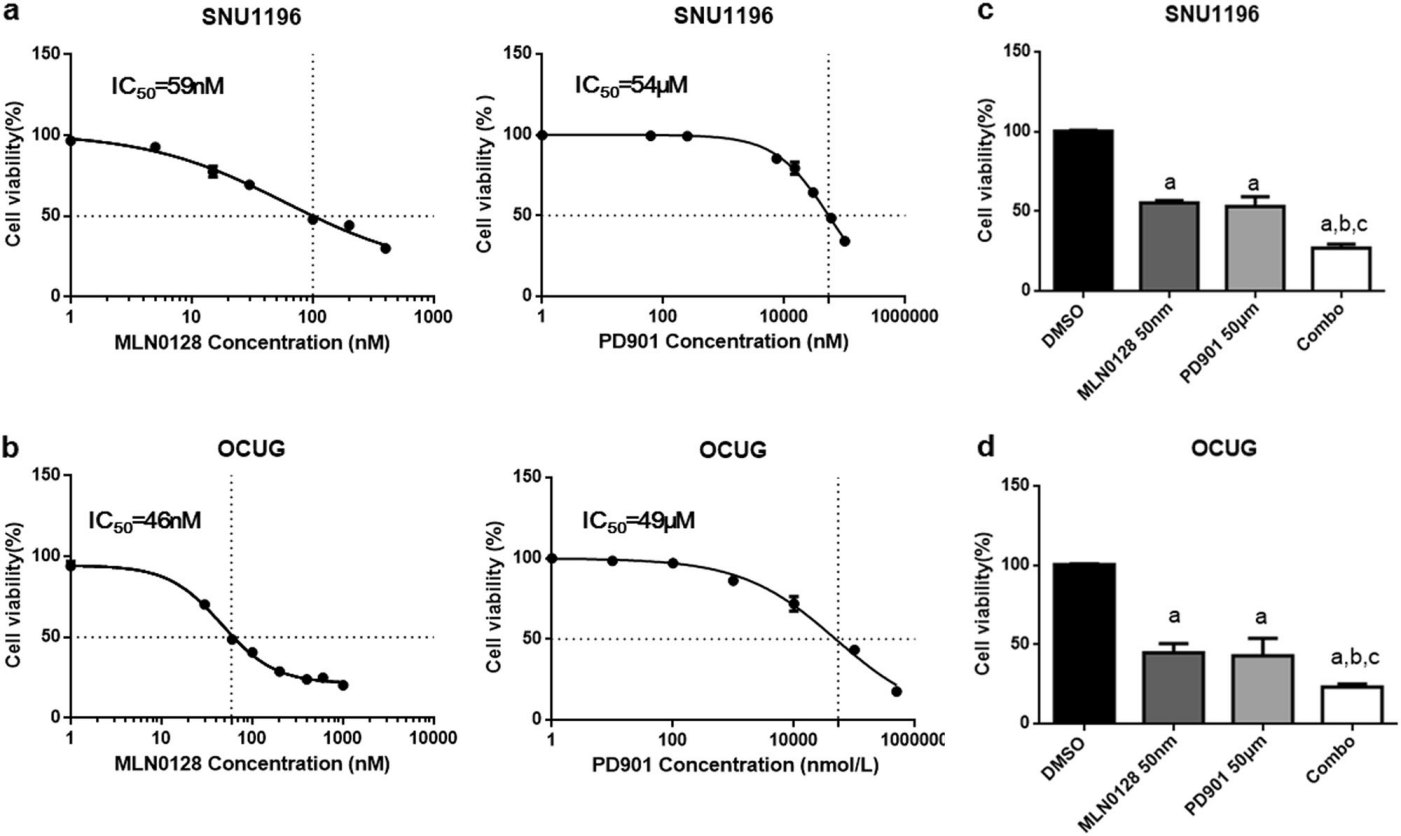
MNL0128 and/or PD901 inhibit *K-Ras* wild-type CCA cell growth in vitro. **a**, **b** IC_50_ values calculated by quantifying the Crystal violet staining from SNU1196 (**a**) and OCUG (**b**) cell line treated for 3 days with the indicated doses of PD901 and MNL0128. **c**, **d** Combining PD901 with MNL0128 (concentration of IC_50_) resulted in reduced cell viability of SNU1196 (**c**) and OCUG (**d**) compared with PD901 or MNL0128 single treatment. Abbreviations: Combo, combined PD9018/MNL012 treatment. Tukey–Kramer test: at least *P* < 0.005 **a**, vs DMEM **b**, vs MLN0128; **c**, vs PD901

**Fig. 2:**
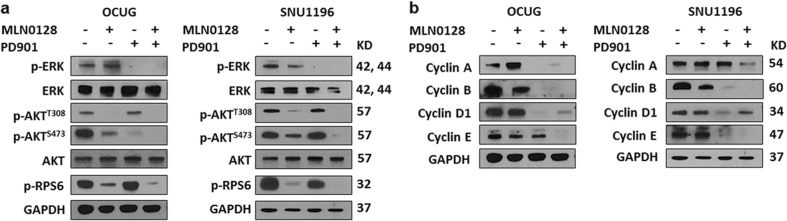
Biochemical analysis of PD901 (50 μM) and/or MLN0128 (50 nM) treatment in *K-Ras* wild-type CCA cell lines. **a** Representative western blot analysis of AKT/mTOR and Ras/MAPK pathway genes in OCUG and SNU1196 cell lines. **b** Representative Western Blot analysis of proliferation signaling pathway genes in OCUG and SNU1196 cell lines

We evaluated the growth inhibitory activity of MEK inhibitor PD901 and pan-mTOR inhibitor MLN0128 in suppressing *K-Ras* wild-type CCA cell growth (Fig. 1). Two cell lines, SNU1196 and OCUG cells, were randomly selected among a panel of *K-Ras* wild-type CCA cell lines. We found that PD901 was able to inhibit SNU1196 and OCUG CCA cell growth with IC_50_ around 50 µM, and MLN0128 was able to inhibit SNU1196 and OCUG cell growth with IC_50_ around 50 nM (Fig. 1a, b). When both PD901 and MLN0128 were added to the culture system, a stronger growth inhibitory effect was observed (Fig. 1c, d). At the biochemical level, as expected, MLN0128 significantly inhibited the expression of mTORC2 targets p-AKT^T308^ and p-AKT^S473^ as well as mTORC1 targets p-RPS6 (Fig. 2a). PD901 remarkably reduced the expression of p-ERK (Fig. 2a). The combination treatment led to a further decreased level of the AKT/mTOR cascade in CCA cell lines (Fig. 2a).

**Fig. 3:**
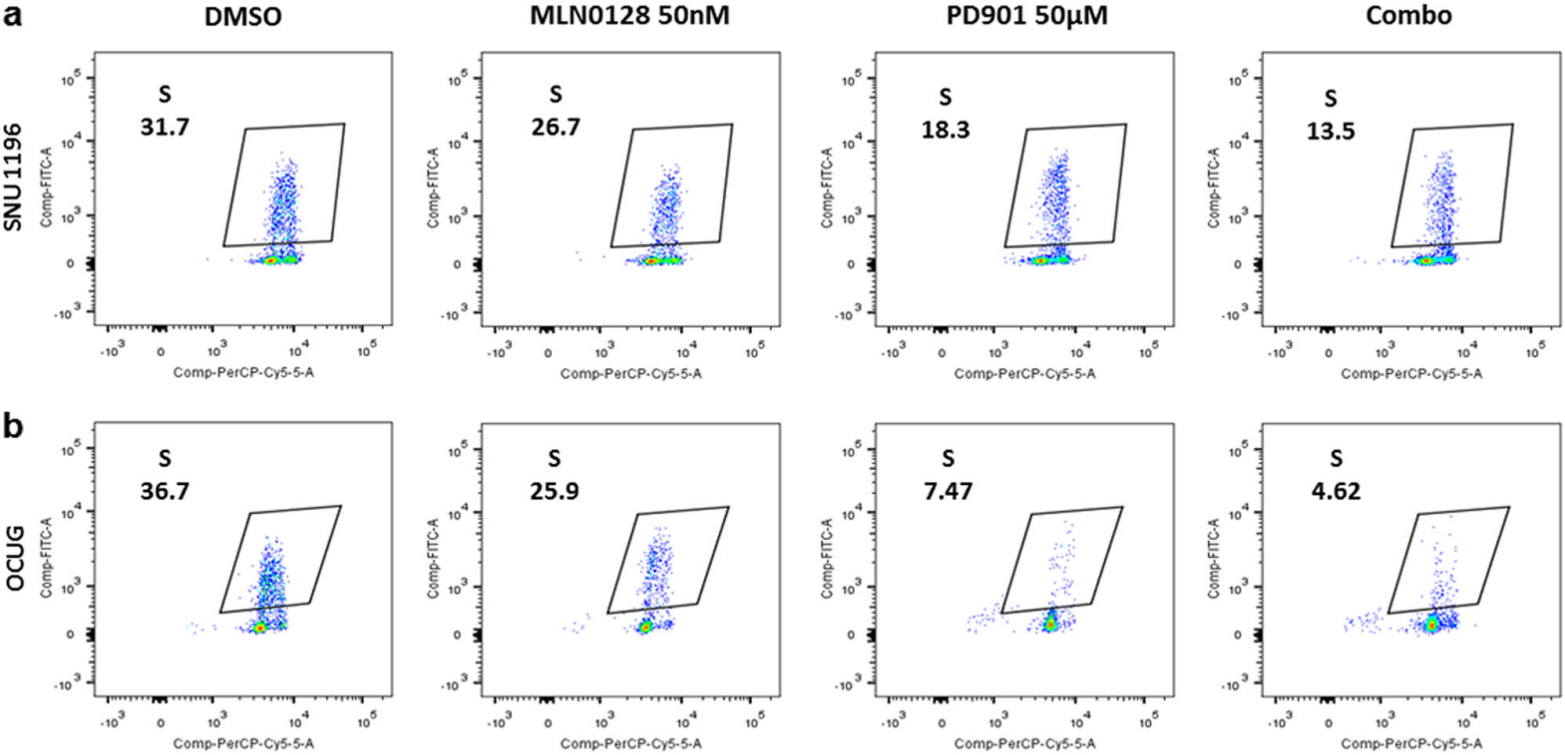
Effect of PD901 (50 μM) and/or MLN0128 (50 nM) on cell cycle progression of CCA cell lines. Effect of PD901 (50 μM) and/or MLN0128 (50 nM) on cell cycle progression of SNU1196 (**a**) and OCUG (**b**) *K-Ras* wild-type type CCA cell lines was analyzed via pulse-labeled with BrdU for 1 h followed by flow cytometric analysis. The percentage of cells in the S phase is shown, together with the representative dot plots. Values are the mean of two experiments. Abbreviations: Combo combined PD901/MLN0128 treatment

In a recent genomic analysis of human CCA samples, it was discovered that CCAs could be classified into two distinct molecular classes. Approximately 40% of CCAs were characterized by the activation of inflammatory signaling pathways, while the remaining 60% CCAs showed a high proliferation rate with predominant activation of oncogenes associated with poor patient outcome, including Ras/MAPK and AKT/mTOR pathways^22^. Therefore, we focused on how Ras/MAPK and AKT/mTOR cascades might control CCA cell cycle progression and tumor proliferation. For this purpose, we treated the panel of CCA cell lines with PD901 and MLN0128, and the IC_50_ values against these inhibitors are summarized in Sup. Table 1. We determined the effect of PD901, either alone or in combination with MLN0128, on proliferation and apoptosis of SNU1196 and OCUG cell lines (Sup. Figure 1). Noticeably, the two drugs affected the growth of SNU1196 and OCUG cell lines via distinct mechanisms. While PD901 mainly restrained cell proliferation with limited effects on apoptosis, MLN0128 strongly induced apoptotic cell death without significantly decreasing cell proliferation (Sup. Figure 1). Concomitant administration of the two drugs led to a stronger anti-growth effect, further reducing proliferation and enhancing apoptosis in SNU1196 and OCUG cells. Subsequently, flow cytometric assay was conducted in the two CCA cell lines treated with PD901 and MLN0128, alone or in combination. Again, treatment with PD901 led to a significantly decreased number of cells in S-phase, whereas MLN0128 administration had only a mild effect on CCA cell cycle progression. Combined treatment with PD901 and MLN0128 showed a further increase in cell cycle arrest (Fig. 3a, b). The effects of PD901 to inhibit *K-Ras* wild-type CCA cell cycle progression was further evaluated in three additional cell lines. Consistently, PD901 strongly inhibited cell cycle progression in all *K-Ras* wild-type CCA cell lines tested (Sup. Figure 2). Next, we analyzed the expression of cell cycle related protein Cyclin A, B, D1, and E in CCA cell lines (Fig. 2b). Consistently, we found that PD9901, but not MLN0128, had a profound effect in inhibiting cell cycle related protein expression (Fig. 2b).

Altogether, these results suggest that the Ras/MAPK cascade, but not the AKT/mTOR pathway, is a major regulator of *K-Ras* wild-type CCA cell proliferation in culture. Combined targeting of Ras/MAPK and AKT/mTOR pathways increases growth inhibitory activity in vitro.

### Therapeutic potential of PD901, alone or in combination with MLN0128, in the AKT/YapS127A CCA preclinical model

Next, we evaluated the therapeutic potential of MEK inhibitors for the treatment of *K-Ras* wild-type CCA in vivo using the AKT/YapS127A CCA preclinical model^19^. As the in vitro experiments indicated that combined MEK and mTOR inhibitors induce strong growth suppression, we applied PD901 alone or in combination with MLN0128 in the preclinical study.

As a first step, we evaluated the maximum dose of PD901 and MLN0128 that could be applied to mice. Previous studies suggest that PD901 at 10 mg/kg/day and MLN0128 at 1 mg/kg/day are well-tolerated in mice. Thus, the drugs were given to the mice for 5 days. Mouse body weight was used as the readout of overall drug toxicities. The results showed that PD901 could be administered at 10 mg/kg/day, while MLN0128 could be dosed at 1 mg/kg/day. However, combination of PD901 at 10 mg/kg/day and MLN0128 at 1 mg/kg/day was highly toxic to the mice. Consequently, we decreased MLN0128 dose to 0.5 mg/kg/day, since we found that, at this lower dose, it could be combined with PD901 at 10 mg/kg/day with limited overall toxicity. Thus, PD901 and MLN0128 at the dose of 10 mg/kg/day and 0.5 mg/kg/day, respectively, were selected for the preclinical study.

**Fig. 4:**
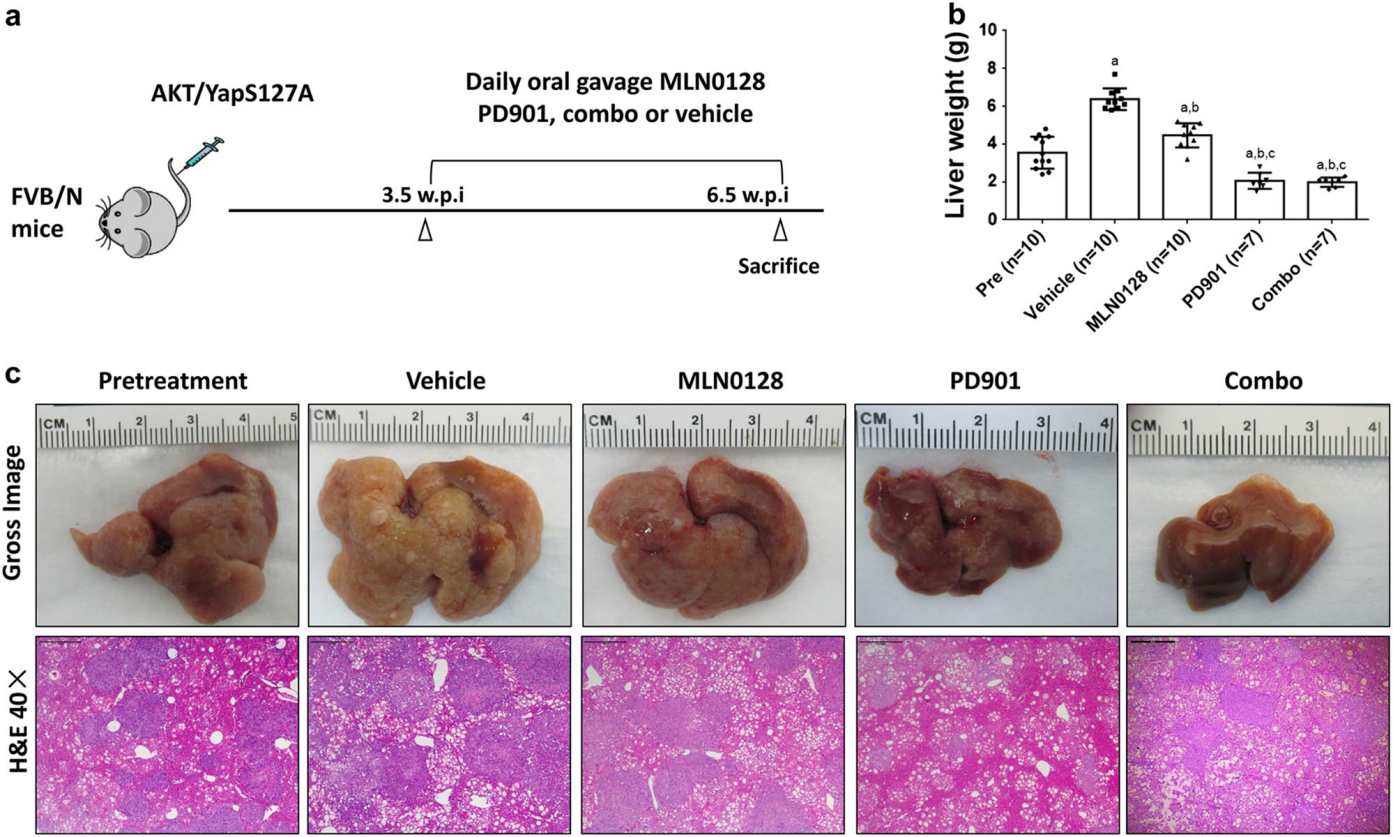
Therapeutic efficacy of PD901 and MLN0128 in AKT/YapS127A preclinical CCA model. **a** Study design. **b** Liver weight of pretreatment, vehicle-, MLN0128-, PD901- and PD901/MLN0128-treated AKT/YapS127A mice. **c** Gross images and H&E staining of AKT/YapS127A mouse liver tissues. Abbreviations: Combo combined PD901/MLN0128 treatment

We hydrodynamically injected mice with AKT/YapS127A and monitored the tumor growth. Specifically, 3.5 weeks after injection, a moderate tumor burden was observed in most mice (average liver weight 3.5 g) (Fig. 4a, b). Mice were then separated into five cohorts. The animals from one group were harvested as pre-treatment cohort and were used as the baseline group for the study. The remaining four groups were treated with vehicle, 10 mg/kg/day PD901, 0.5 mg/kg/day MLN0128, or 10 mg/kg/day PD901 plus 0.5 mg/kg/day MLN0128 combination. Mice were euthanized if they became moribund or developed large abdominal mass, and therefore were considered “deceased” based on our IACUC protocol. All surviving mice were harvested after 3 weeks of drug treatment (Fig. 4a). We used total liver weight as the measurement of tumor burden.

As shown in Fig. 4b, the vehicle group showed the highest tumor burden. MLN0128 groups exhibited significant lower tumor burden than vehicle cohort but higher than the pre-treatment group, suggesting the tumors continued to grow despite of MLN0128 administration. PD901 monotherapy and PD901/MLN0128 combination therapy groups displayed lower tumor burden even when compared with pretreatment group. PD901/MLN0128 combination therapy group did not show a lower tumor burden when compared to PD901 monotherapy (Fig. 4b).

**Fig. 5:**
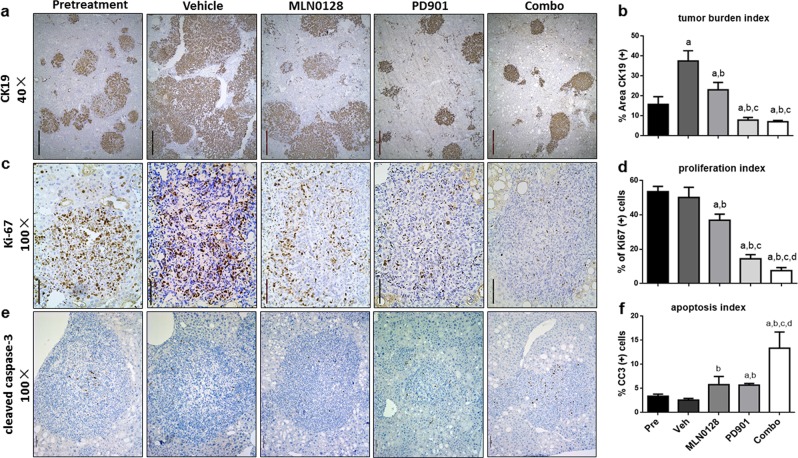
Effects of PD901 and/or MLN0128 on AKT/YapS127A CCA tumor cell proliferation and apoptosis. **a** CK19, **c** Ki-67, and **e** Cleaved caspase 3 (CC3) staining in livers from AKT/YpS127A mice subjected to the various treatments. **b** CK19 staining was quantified as the percentage of the positive staining area of the whole section area and used as tumor burden index. **d** Ki-67 positive cells were counted and quantified as proliferation index. **e** Cleaved caspase 3 (CC3) positive cells were counted and quantified as apoptosis index. Tukey–Kramer test: at least *P* < 0.001. **a**, vs Pretreatment; **b**, vs Vehicle; **c**, vs MLN0128; **d**, vs PD901; **e**, vs Combo. Abbreviations: Pre pretreatment, Veh vehicle, Combo combined PD901/MLN0128 treatment

Hematoxylin and eosin staining revealed that the tumor type in all groups was CCA (Fig. 4c), which was further confirmed by the diffuse immunoreactivity for the biliary marker CK19 (Fig. 5a). Consistently, using percentage of CK19(+) area as a second approach for measuring tumor burden, PD901 treatment led to tumor shrinkage, with combined PD901/MLN0128 therapy not providing further benefit (Fig. 5b).

In summary, the in vivo experiments indicate that PD901 treatment induces tumor regression in the AKT/YapS127A preclinical CCA mouse model. Combined PD901/MLN0128 treatment does not provide additional advantages to PD901 monotherapy.

### PD901 regimen strongly inhibits tumor cell proliferation in vivo

To evaluate proliferation status in the five mouse cohorts, Ki-67 immunohistochemistry was employed (Fig. 5c). The results indicated that MLN0128 treatment mildly inhibited tumor cell proliferation. In striking contrast, PD901 and combination therapy significantly reduced CCA cell proliferation rates in vivo (Fig. 5d). Subsequently, we analyzed the apoptosis indexes using cleaved caspase-3 (CC3) immunohistochemistry as a marker of programmed cell death (Fig. 5e). Overall, few CC3(+) cells were detected in CCAs, with the highest number of these cells being observed in PD901/MLN0128 treated mice (Fig. 5f).

**Fig. 6:**
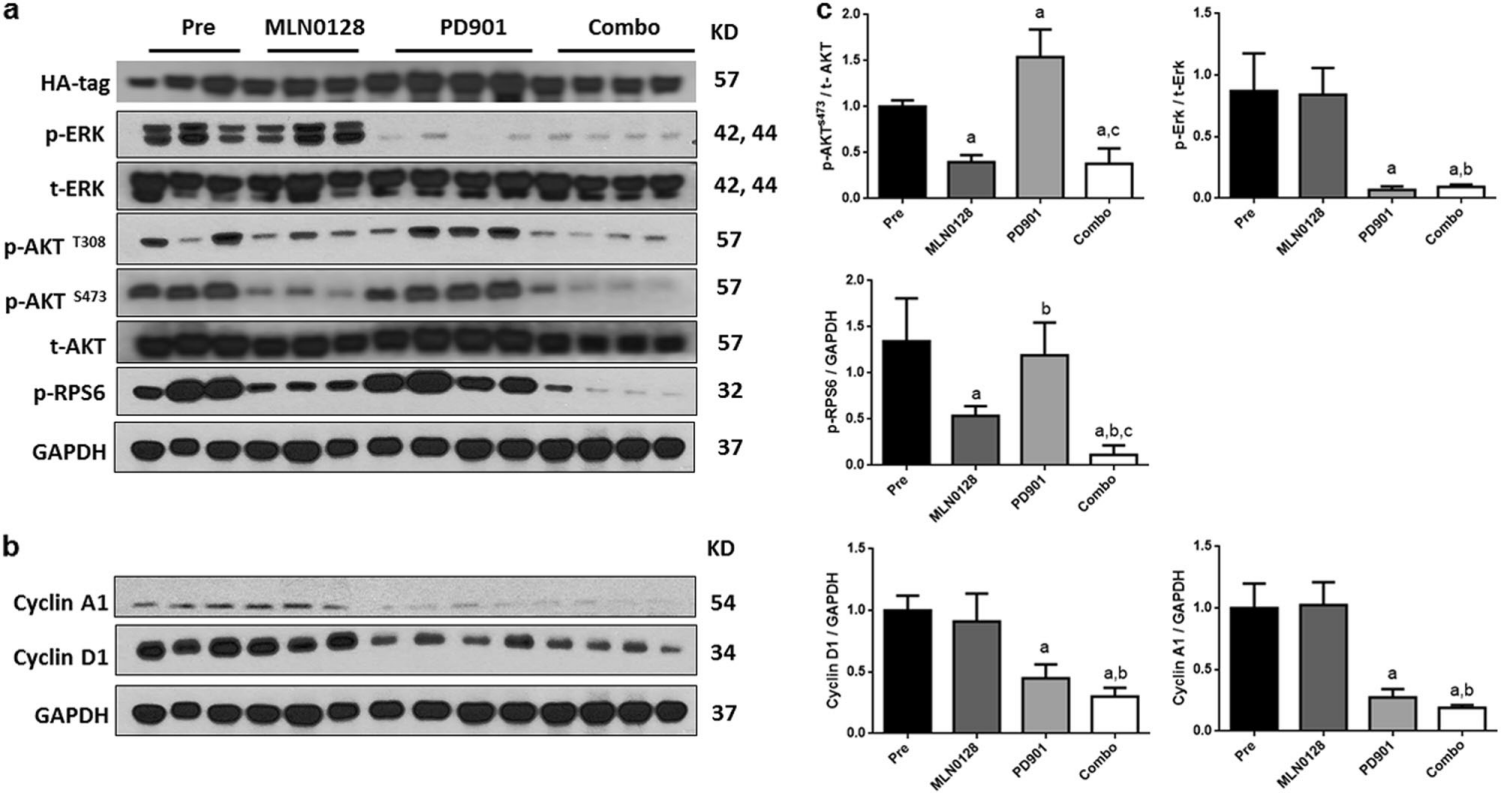
Biochemical analysis of PD901 and/or MLN0128 on AKT/YapS127A CCA tumor cells. Western blot analysis was performed to analyze AKT/mTOR and Ras/MAPK pathways (**a**) and cell proliferation (**b**) in CCA tissues from pretreatment, vehicle-, MLN0128-, PD901- and PD901/MLN0128-treated AKT/YapS127A mice. HA-tag was used to normalize tumor burden among different cohorts; and GAPDH was used as loading control. **c** Quantified analysis of target proteins. Abbreviations: Pre, pretreatment, Combo combined PD901/MLN0128 treatment

At the molecular level, p-ERK1/2 expression was completely abolished in PD901-treated mice (Fig. 6a and Sup. Figure 3), while MLN0128 treatment led to decreased p-AKT and p-RPS6 levels (Fig. 6a). Combined PD901/MLN0128 administration triggered the inhibition of both AKT/mTOR and Ras/MAPK cascades (Fig. 6a, c). Next, we focused on the expression of cell cycle related genes. Noticeably, PD901, but not MLN0128, inhibited the expression of Cyclin A1 and Cyclin D proteins in mouse CCA tissues (Fig. 6b, c).

In summary, our findings demonstrate that in the *K-Ras* wild-type AKT/YapS127A CCA preclinical model, PD901 treatment results in tumor regression via strong inhibition of tumor cell proliferation.

### PD901 but not MLN0128 treatment modulates tumor microenvironment in AKT/YapS127A mice

Tumor microenvironment has been shown to play a major role in tumor initiation and progression. The use of the preclinical murine model enabled us to analyze how the Ras/MAPK and AKT/mTOR signaling modulate tumor microenvironment, which could not be studied using in vitro tumor cell culture.

**Fig. 7:**
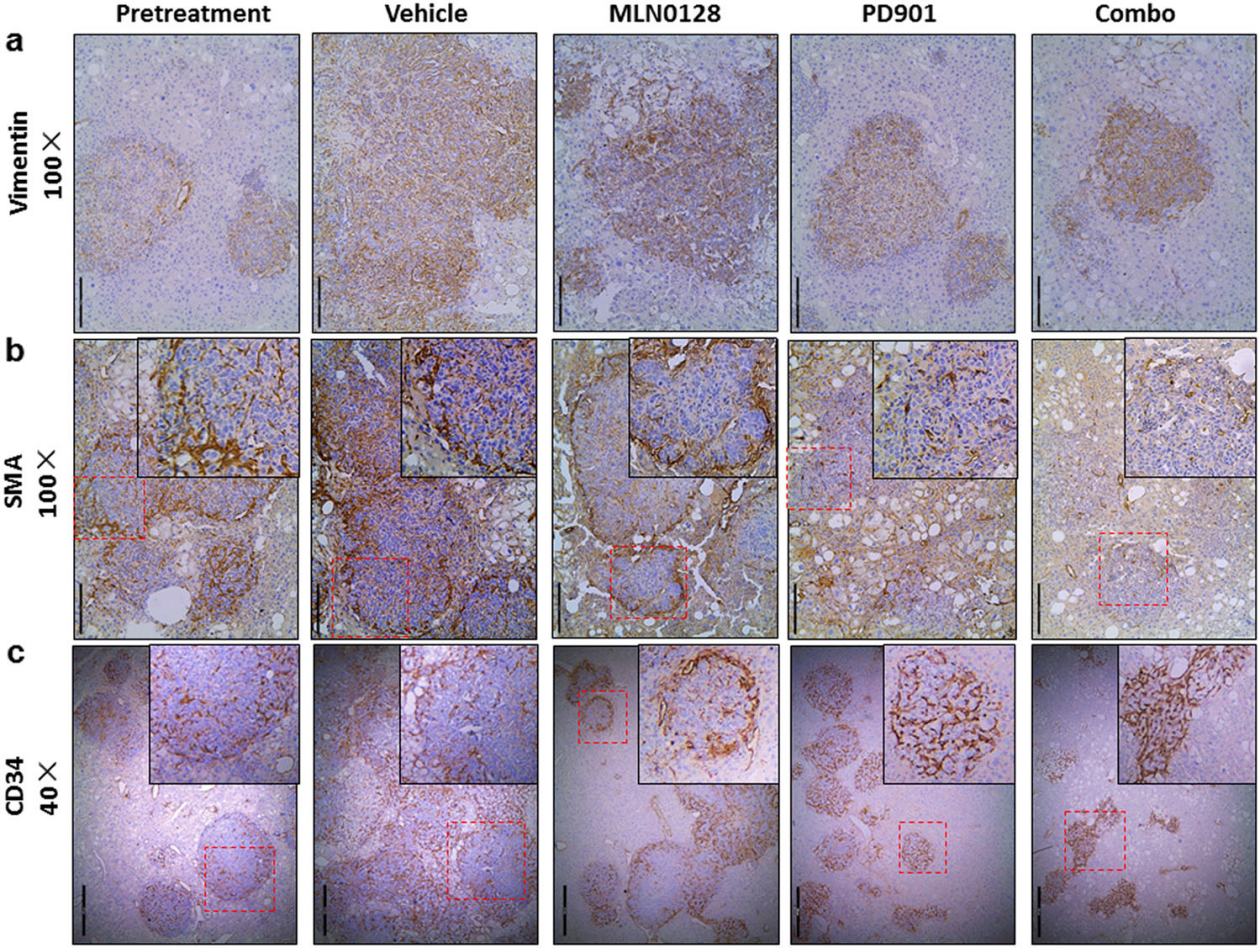
Effects of treatment with PD901 and MLN0128, either alone or combination, on the tumor microenvironment of AKT/YapS127A CCA nodules, as illustrated by immunohistochemistry. **a** Vimentin, **b** SMA, and **c** CD34 staining in livers from AKT/YapS127A mice subjected to the various treatments. Abbreviations: Combo combined PD901/MLN0128 treatment, SMA α-smooth muscle Actin. Insets: expanded views of the area indicated by red boxes

Human CCAs are characterized by the presence of prominent and hypovascularized stromal cells; these cells, known as cancer-associated fibroblasts (CAFs), have an important role on cholangiocarcinogenesis^23,24^. In our previous study, we demonstrated that AKT/YapS127A CCA model recapitulates this phenotype^19^. CAFs can be classified as vimentin (+) fibroblasts, which infiltrate the tumor (Fig. 7a); and smooth muscle actin (SMA) (+) myofibroblasts, which predominantly form a capsule-like structure surrounding each CCA nodule (Fig. 7b). PD901 and/or MLN0128 treatment did not affect the overall vimentin (+) CAF patterns (Fig. 7a). In contrast, PD901, but not MLN0128 treatment, led to a profound loss of SMA(+) CAFs in CCA nodules. While SMA(+) CAFs surrounding tumor nodules in pretreatment, vehicle or MLN0128 treated CCA were clearly appreciable, few SMA(+) CAFs could be seen surrounding the CCA nodules in PD901 and PD901/MLN0128 mouse liver tissues (Fig. 7b). Since PD901 appears to strongly modulate CAFs, we analyzed additional CAF markers, including hyaluronic acid (HA) and S100A4, in control and PD901 treated CCA samples. We found that HA stained CAFs in a similar pattern as vimentin, and it was not affected by PD901 (Sup. Figure 4a). S100A4(+) CAFs could be found scattered in the CCA lesions in pretreatment mice. S100A4(+) CAFs decreased during tumor progression, as less S100A4(+) CAFs could be detected in vehicle treated mice. In PD901 treated mice, scattered S100A4(+) CAFs were observed, similar to that in pretreatment mice (Sup. Figure 4B). The results add additional evidence supporting a major role played by the Ras/MAPK cascade in regulating CAFs.

Angiogenesis is a fundamental feature of tumor microenvironment, which is responsible for the vascular network formation and the recruitment of inflammatory cells^25^. Imaging studies for human CCA suggest that most of mass-forming CCAs manifest as hypovascular mass with a hypervascular rim^26^. This phenotype was clearly recapitulated in AKT/YapS127A murine CCA, as demonstrated by CD34 immunostaining, showing that endothelial cells were predominantly located in the outer layers of CCA tumor nodules (Fig. 7c). Few endothelial cells could be found in the center of the nodules (Fig. 7c). Of note, MLN0128 did not affect the vasculature of tumor nodules, whereas in the PD901 and combination treatment groups, CD34 (+) endothelial cells could be found scattered inside the tumor tissue (Fig. 7c).

The significant modulation of tumor microenvironment characterized by the loss of SMA(+) CAFs and increased CD34(+) vasculature in PD901 treated CCA may profoundly affect oxygen supplies to the tumor cells. It is known that hypoxia may enhance the aggressiveness of CCA cells^27^. To study the hypoxia status in mouse CCA, additional AKT/YapS127A CCA bearing mice were generated, treated with vehicle or PD901. Hypoxyprobe was injected into the mice before they were harvested (Sup. Figure 5a). We found that weak but appreciable hypoxyprobe staining could be detected in pretreatment AKT/YapS127A tumor nodules. With the increasing tumor size, all vehicle treated mice displayed strong hypoxyprobe staining within the center of the tumor nodules (Sup. Figure 5a). In striking contrast, no hypoxyprobe staining could be found in PD901 treated CCA nodules (Sup. Figure 5a). Consistent with this observation, nuclear Hif-1α, the central regulator of hypoxia in cells, could be easily found in pretreatment and vehicle treated AKT/YapS127A CCA tumor cells, but not in PD901 treated tumor cells (Sup. Figure 5b). The decreased expression of Hif-1α and its downstream effectors, including HK1, HK2, PKM1, and PKM2 in PD901 treated tumor cells was further confirmed by Western blotting (Sup. Figure 6). The results suggest the overall decreased hypoxia after MEK inhibitor treatment in CCA.

In conclusion, our observations indicate that in AKT/YapS127A mice, PD901 but not MLN0128 treatment is able to modulate tumor microenvironment though remodeling CAFs and vasculature, leading to decreased hypoxia.

## Discussion

In our current study, we investigated the functional role of the Ras/MAPK cascade in regulating *K-Ras* wild-type CCA development and evaluated the therapeutic potential of a MEK inhibitor, either alone or in combination with an AKT/mTOR inhibitor, for CCA treatment. Our results show that Ras/MAPK, but not AKT/mTOR pathway, is the prominent regulator of cell cycle progression in *K-Ras* wild-type CCA cells in culture and in mice. In particular, in the AKT/YapS127A preclinical murine CCA model, MEK inhibitor PD901 treatment for 3 weeks led to over 70% of decrease of Ki-67 index. This is in contrast to our recent study on MEK inhibitor for the treatment of *K-Ras* mutant CCA^10^. In the K-Ras/NICD CCA model (harboring *K-Ras* mutant), indeed, treatment with PD901 at the same dose for 3 weeks did not affect tumor cell proliferation, as indicated by the similar Ki-67 indexes in vehicle and PD901 treated CCA samples^10^. At the molecular level, in AKT/YapS127A mice, PD901 treatment resulted in the decreased expression of cell cycle proteins, including Cyclin A1 and cyclin D (Fig. 6b), whereas in K-Ras/NICD CCA model, PD901 treatment did not significantly alter the levels of these proteins^10^. Together, these results suggest that in *K-Ras* wild-type CCA, Ras/MAPK cascade regulates mainly tumor cell proliferation, and MEK inhibitor treatment leads to tumor regression due to a strong induction of cell cycle arrest. In *K-Ras* mutant CCA, Ras/MAPK cascade might instead modulate tumor cell survival, with MEK inhibitor treatment leading to stable disease due to increased apoptosis. Clearly, additional experiments are required to elucidate the molecular mechanisms underlying this observation and to characterize how Ras/MAPK cascade regulates signaling pathways promoting cell proliferation and survival under different genetic backgrounds.

Another important conclusion from our study is that Ras/MAPK pathway plays a major role in modulating tumor microenvironment in CCA. It is important to note that studies of tumor microenvironment are particularly challenging using in vitro cell culture systems. Therefore, the use of animal models is critical to study how a pathway or drug treatment might function to modulate tumor microenvironment. In our study, we discovered that the MEK inhibitor, but not the mTOR inhibitor leads to the decrease of SMA(+) CAFs. Previous studies have demonstrated that SMA(+) CAFs promote CCA growth in vitro and in vivo^28,29^, with the expression of SMA(+) CAFs being correlated with the poor prognosis of human CCA patients^29,30^. It has been speculated that SMA(+) CAFs are derived from activated hepatic stellate cells^31^. Our study therefore suggests that the Ras/MAPK cascade has a critical role in inducing the proliferation as well as the recruitment of activated hepatic stellate cells surrounding CCA tumors.

In addition, we also noted that MEK inhibition led to the increased vasculature in tumor nodules, effectively decreasing tumor hypoxia. Hypoxia is one of the key features of human CCA^32^ and has been shown to enhance CCA cell proliferation^33,34^. We also investigated whether PD901 may directly affect HIF-1α expression in tumor cells. Towards this goal, we treated OCUG and SNU1196 cells with dimethyloxaloylglycine (DMOG), a HIF-1α inducer, either alone or in combination with PD901. We discovered that PD901 treatment effectively inhibited DMOG induced HIF-1α expression in both CCA cell lines (Sup. Figure 7). Together these results suggest that MEK inhibitors may suppress hypoxia status in CCAs directly via inhibiting HIF-1α expression in CCA cells as well as indirectly via modulating tumor microenvironment. Positive expression of Hif-1α has been reported to be an independent prognostic factor for CCA patient survival^33^. Our results therefore indicate that Ras/MAPK signaling is a major regulator of hypoxia in CCA.

It is worth to note that both decreased CAFs and decreased tumor hypoxia may lead to decreased tumor cell proliferation. Therefore, it is likely that the profound growth arrest phenotype induced by MEK inhibitors in *K-Ras* wild-type CCA may be due to multiple mechanisms, including the intrinsic dependence of CCA cells on Ras/MAPK cascade for cell proliferation, and altered microenvironment, leading to additional suppression of cell proliferation.

In this study, we showed that, in vitro, treatment of CCA cells with combined PD901 and MLN0128, each at IC_50_ concentration, results in a stronger growth inhibition when compared with treatment with both drugs administered alone. In contrast, in AKT/YapS127A mice, combined treatment with PD901 and MLN0128 did not lead to further decrease in tumor burden when compared to PD901 treatment alone. This might be at least partially due to the fact that while mice could tolerate MLN0128 at 1 mg/kg/day in monotherapy, MLN0128 had to be decreased to 0.5 mg/kg/day when the two drugs were combined. Consequently, the decreased efficacy by MLN0128 when compared with our previous report might be concentration-dependent^19^. It is worth to note that combined PD901 and MLN0128 treatment in AKT/YapS127A mouse lesions triggered a further decrease in cell proliferation and increase in apoptosis, although both effects were relatively mild when compared to those achieved by monotherapies. It is still possible that, over long term, the combined treatment may show additional survival benefit. Nevertheless, one needs to balance the survival benefit and possible increased toxicity induced by the combination therapy during cancer treatment. Additional studies are required to further investigate whether combined treatment with MEK and mTOR inhibitors may be superior to MEK inhibitor alone for the treatment for *K-Ras* wild-type CCA.

Our investigation contains multiple translation implications. As Ras/MAPK signaling cascade has been shown to be almost ubiquitously activated in human CCAs^10^, the present findings support the further evaluation of MEK inhibitors for CCA treatment in clinical trials. In our preclinical study, MEK inhibitor alone did not completely eliminate tumor cells in mice. This may be due to the fact that it has limited effects in inducing tumor cell apoptosis. The combination of MEK inhibitors with drugs with strong pro-apoptotic activity, such as the selective BCL-2 inhibitor Venetoclax/ABT-199, might be helpful for enhanced therapeutic efficacy.

## Materials and methods

### Constructs and reagents

The constructs used for mouse injection, including pT3-EF1α, pT3-EF1α-HA-myr-AKT (mouse), pT3-EF1α-YapS127A (human), and pCMV/sleeping beauty transposase (pCMV/SB) plasmids, were described previously^35–37^. Plasmids were purified using the Endotoxin free Maxi Prep Kit (Sigma-Aldrich, St. Louis, MO) before being injected into mice. PD0325901 (PD901) and MLN0128 were purchased from LC Laboratories (Woburn, MA). PD901 and MLN0128 were dissolved in DMSO for in vitro experiments.

### Hydrodynamic tail vein injection and mouse treatment

Female wild-type (WT) FVB/N mice were obtained from Charles River Laboratories (Wilmington, MA). Hydrodynamic injection was performed as described previously in detail^38^. To induce CCA in mice, 20 μg pT3-EF1α-HA-myr-AKT and 30 μg pT3-EF1α-YapS127A and 2 μg pCMV/SB were injected in FVB/N mice. MLN0128 (0.5 mg/kg/day), PD901 (10 mg/kg/day), MLN0128 + PD901 or vehicle were orally administered via gavage. Therapy administration began 3.5 weeks post injection for 3 consecutive weeks, and mice were sacrificed 6.5 weeks after hydrodynamic injection. MLN0128 was dissolved in 1-methyl-2-pyrrolidinone (NMP; Sigma) to make a stock solution of 20 mg/ml and the aliquots were stored at −80 °C. It was diluted 1:200 into 15% PVP/H2O (PVP: polyvinylpyrrolidone K 30, Sigma-Aldrich; diluted in H_2_O at a 15.8:84.2 w/v ratio). PD901 was dissolved in 0.5% (w/v) hydroxypropyl-methylcellulose (HPMT; Sigma) in water plus 0.2% v/v Tween 80 to a stock concentration of 3.33 mg/ml. PD901 was orally administered via gavage for 5 days/week. Before gavage, stock solution was diluted with 0.9% NaCl to make a microemulsion. Mice were housed, fed, and monitored in accord with protocols approved by the Committee for Animal Research at the University of California San Francisco (San Francisco, CA).

### Measurement of hypoxia in vivo

In vivo hypoxia status of mouse CCA tumors was analyzed using a separate cohort of mice collected at 3.5 weeks post injection (pretreatment), 3 weeks treatment with vehicle or 3 weeks treatment with PD901 (10 mg/kg/day) using Hypoxyprobe^TM^-1 kit (Burlington, MA). In brief, pimonidazole hydrochloride marker, i.e. Hypoxyprobe^TM^-1, was injected intraperitoneally (60 mg/kg) into each mouse 60 min prior to euthanasia. Liver tissue were harvested, formalin fixed, and embedded in paraffin. Detection of HyproxyprobeTM-1 was performed using immunohistochemical staining, following the manufacturer’s protocol.

### Histology and immunohistochemistry

Mouse liver specimens were fixed overnight in 4% paraformaldehyde (Anatech Ltd.) and embedded in paraffin. Sections were done at 5 μm in thickness. Preneoplastic and neoplastic liver lesions were assessed by two board-certified pathologists and liver experts (M.E. and K.U.). For immunohistochemical staining, slides were deparaffinized in xylene, rehydrated through a graded alcohol series, and rinsed in PBS. Antigen retrieval was performed either in 10 mM sodium citrate buffer (pH 6.0) or in 1 mM ethylenediaminetetraacetic acid (EDTA; pH 8.5) by placement in a microwave oven on high for 10 min, followed by a 20-min cool down at room temperature. After a blocking step with the 5% goat serum and Avidin-Biotin blocking kit (Vector Laboratories, Burlingame, CA), the slides were incubated with primary antibodies (Sup. Table 2) overnight at 4 °C. Slides were then subjected to 3% hydrogen peroxide for 10 min to quench endogenous peroxidase activity and, subsequently, the biotin conjugated secondary antibody was applied at a 1:500 dilution for 30 min at room temperature. The immunoreactivity was visualized with the Vectastain Elite ABC kit (Vector Laboratories, Burlingame, CA) and 3,3′- diaminobenzidine as the chromogen. Slides were counterstained with hematoxylin. Proliferation and apoptosis indices were determined in mouse CCA lesions by counting Ki-67 and Caspase 3 positive cells, respectively, on at least 3000 tumor cells per mouse sample. HA was stained using biotin-labeled HA-binding protein as previously described^39^. After being dewaxed and rehydrated, endogenous peroxidase was blocked with 3% H_2_O_2_ for 10 min, followed by Avidin/Biotin blocking. Liver sections were incubated with 4 μg/ml biotin-labeled HA-binding protein (rhAggrecan aa20-675/His (NSO/7), biotin, R&D Systems) for 2.5 h. After washing with PBS, VECTASTAIN Elite ABC kit was applied. HA was visualized with DAB peroxidase substrate kit (Cat. PK-6100 and SK-4100, Vector Laboratories) and tissues were counterstained with hematoxylin.

### Western blot analysis

Frozen mouse livers tissues and cultured cell samples were homogenized in lysis buffer, consisting of 30 mM Tris (pH 7.5), 150 mM NaCl, 1% NP-40, 0.5% Na deoxycholate, 0.1% SDS, 10% glycerol and 2 mM EDTA, and containing the Complete Protease Inhibitor Cocktail (ThermoFisher Scientific, Waltham, MA). Protein concentrations were determined with the Bio-Rad Protein Assay Kit (Bio-Rad, Hercules, CA) using bovine serum albumin as standard. For Western blot analysis, aliquots of 40 μg were denatured by boiling in Tris-Glycine SDS Sample Buffer (Bio-Rad), separated by SDS-PAGE, and transferred onto nitrocellulose membranes (Bio-Rad) by electroblotting. Membranes were blocked in Pierce Protein-free Tween 20 Blocking Buffer (ThermoFisher Scientific) for 1 h and probed with specific antibodies listed in Sup. Table 2. Anti-β-Actin (Sigma-Aldrich) or GAPDH (EMD Millipore, Burlington, MA) antibody was used as loading control. Each primary antibody was followed by incubation with horseradish peroxidase-secondary antibody (Jackson Immunoresearch Laboratories Inc., West Grove, PA) diluted 1:5000 for 30 min and proteins were revealed with the Super Signal West Femto (Pierce Chemical Co., New York, NY).

### In vitro experiments

*K-Ras* wild-type human CCA cell lines, including SNU1196, OCUG, KMCH, MzCha and Huh28, were used for the in vitro studies. Cell lines were maintained as monolayer cultures in Dulbecco’s modified Eagle medium supplemented with 10% fetal bovine serum (FBS; Gibco, Grand Island, NY, USA), 100 U/ml penicillin, and 100 g/ml streptomycin (Gibco, Grand Island, NY, USA). For IC_50_ determination, cells were seeded in 24-well plates and treated with increasing doses of PD901 in triplicate for 24–48 h. Cells were stained with crystal violet. After washing, stained cells were incubated in lysis solution and shaken gently on a rocking shaker for 20–30 min. Diluted lysate solutions were added to 96-well plates and OD was measured at 590 nm with a BioTek ELx808 Absorbance Microplate Reader (BioTek Instruments Inc., Winooski, VT). Cell proliferation and apoptosis were assessed in SNU1196 and OCUG cell lines at 24-, 48- and 72-hour time points using the BrdU Cell Proliferation Assay Kit (Cell Signaling Technology, Danvers, MA) and the Cell Death Detection Elisa Plus Kit (Roche Molecular Biochemicals, Indianapolis, IN), respectively, following the manufacturers’ instructions. For the BrdU incorporation assay, control or drug-treated cells were incubated with bromodeoxyuridine (BrdU) for 1 h and the assay was performed using the FITC BrdU Flow Kit (BD Biosciences, San Jose, CA), following the manufacturer’s instructions. The measurement of cell cycle parameters was performed with the Becton Dickinson LSRII Flow Cytometer (BD Biosciences) and the data processed using the FlowJo 10 (FlowJo, LLC, Ashland, OR). All cell line experiments were repeated at least three times in triplicate.

### DMOG-induced hypoxia cell model

OCUG and SNU1196 cells cultured in a humid atmosphere (5% CO_2_) with high glucose DMEM supplemented with 10% FBS at 37 °C were treated with 0.5 mM DMOG (Sigma-Aldrich, St. Louis, MO) for 24 h to induce hypoxia. As for PD901 treatment, 50 μM PD901 were added to cultured cells 1 h before DMOG treatment. After reoxygenation for 24 h, cells were harvested for protein extraction.

### Statistical analysis

GraphPad Prism version 6.0 (GraphPad Software Inc., La Jolla, CA) was used to evaluate statistical significance by Tukey–Kramer test. Data are presented as Means ± SD. *P* values < 0.05 were considered statistically significant.

## Supplementary information

Supplementa tables

Supplemental Figures
