# Supplementary material for: MEK inhibition suppresses K-Ras wild-type cholangiocarcinoma in vitro and in vivo via inhibiting cell proliferation and modulating tumor microenvironment
Source: Cell Death Dis. 2019 Feb 11;10(2):120. doi: 10.1038/s41419-019-1389-4 (PMC6370758; doi:10.1038/s41419-019-1389-4)
Supplement: Supplementary file 1 — Supplementa tables [file 41419_2019_1389_MOESM1_ESM.docx]

**Supplemental Table 1. IC50 against PD901 and MLN0128 in a panel of human CCA cell lines.**

| Cell lines | PD901 IC_50_(μM) | MLN018 IC_50_(nM) |
| --- | --- | --- |
| SNU1196 | 54 | 59 |
| OCUG | 49 | 46 |
| HuCCT1 | 6 | 21[^1^](#_ENREF_1) |
| KKU213 | 6 | 60[^1^](#_ENREF_1) |
| MzCha-1 | 8 | 10[^1^](#_ENREF_1) |
| KMCH | 97 | 16[^1^](#_ENREF_1) |
| RBE | 0.1 | 17[^1^](#_ENREF_1) |

**References**

1. Zhang S*, et al.* Pan-mTOR inhibitor MLN0128 is effective against intrahepatic cholangiocarcinoma in mice. *Journal of hepatology* 2017, **67**(6)**:** 1194-1203.

**Supplemental Table 2. Primary Antibodies for IHC and WB**

| **IHC** | **Concentration** | **Company** | **Catalogue No.** |
| --- | --- | --- | --- |
| α-smooth muscle Actin | 1:400 | DAKO | M0851 |
| CD34 | 1:2500 | Abcam | Ab81289 |
| CK19 | 1:500 | Abcam | Ab133496 |
| Cleaved caspase 3 | 1:100 | Cell Signaling Technology | 9664 |
| F4/80 | 1:200 | Thermo Fisher Scientific | 14-4801-85 |
| HIF-1a | 1:50 | Abcam | AB1 |
| Ki67 | 1:150 | Thermo Fisher Scientific | RM-9106-S1 |
| Phospho-ERK | 1:100 | Cell Signaling Technology | 4370 |
| S100A4 | 1:500 | Cell Signaling Technology | 13018S |

| **WB** | **Concentration** | **Company** | **Catalogue No.** |
| --- | --- | --- | --- |
| AKT | 1:1000 | Cell Signaling Technology | 9272 |
| Phospho-AKTS308 | 1:1000 | Cell Signaling Technology | 13038 |
| Phospho-AKTS473 | 1:100 | Cell Signaling Technology | 3787 |
| Phospho-ERK | 1:100 | Cell Signaling Technology | 4370 |
| Cleaved caspase 3 | 1:500 | Cell Signaling Technology | 9664 |
| Cleaved caspase 7 | 1:500 | Cell Signaling Technology | 5625 |
| Cyclin A | 1:500 | Santa Cruz Biotechnology | SC-751 |
| Cyclin B1 | 1:500 | Santa Cruz Biotechnology | SC-245 |
| Cyclin D1 | 1:5000 | Cell Signaling Technology | 2978 |
| Cyclin E | 1:400 | Biolegend | 630701 |
| ERK | 1:1000 | Cell Signaling Technology | 9102 |
| Phospho-ERK | 1:1000 | Cell Signaling Technology | 9101 |
| HA-Tag | 1:1000 | Cell Signaling Technology | 2367 |
| mTOR | 1:500 | Cell Signaling Technology | 2983 |
| Phospho-4E-BP1(Thr37/46) | 1:1000 | Cell Signaling Technology | 2855 |
| Phospho-4E-BP1(Ser65) | 1:1000 | Cell Signaling Technology | 9451 |
| Phospho-RPS6 | 1:1000 | Cell Signaling Technology | 4858 |
| β-Actin | 1:4000 | Sigma-Aldrich | A5441 |
| GAPDH | 1:5000 | EMD Millipore | MAB374 |
