# Supplementary material for: MEK inhibition suppresses K-Ras wild-type cholangiocarcinoma in vitro and in vivo via inhibiting cell proliferation and modulating tumor microenvironment
Source: Cell Death Dis. 2019 Feb 11;10(2):120. doi: 10.1038/s41419-019-1389-4 (PMC6370758; doi:10.1038/s41419-019-1389-4)
Supplement: Supplementary file 2 — Supplemental Figures [file 41419_2019_1389_MOESM2_ESM.pptx]

## Slide 1
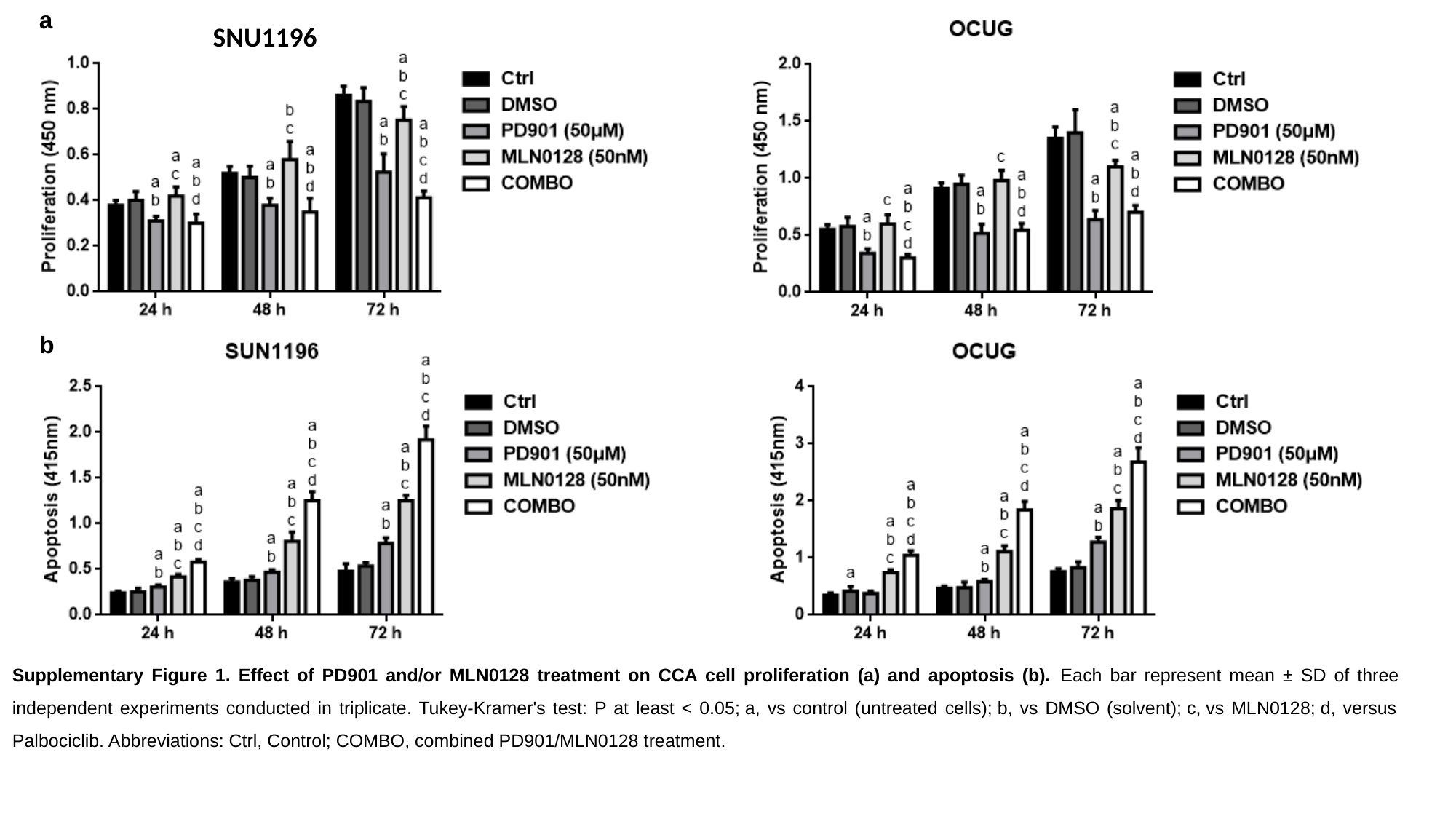

SNU1196
a
b
Supplementary Figure 1. Effect of PD901 and/or MLN0128 treatment on CCA cell proliferation (a) and apoptosis (b). Each bar represent mean ± SD of three independent experiments conducted in triplicate. Tukey-Kramer's test: P at least < 0.05; a, vs control (untreated cells); b, vs DMSO (solvent); c, vs MLN0128; d, versus Palbociclib. Abbreviations: Ctrl, Control; COMBO, combined PD901/MLN0128 treatment.

## Slide 2
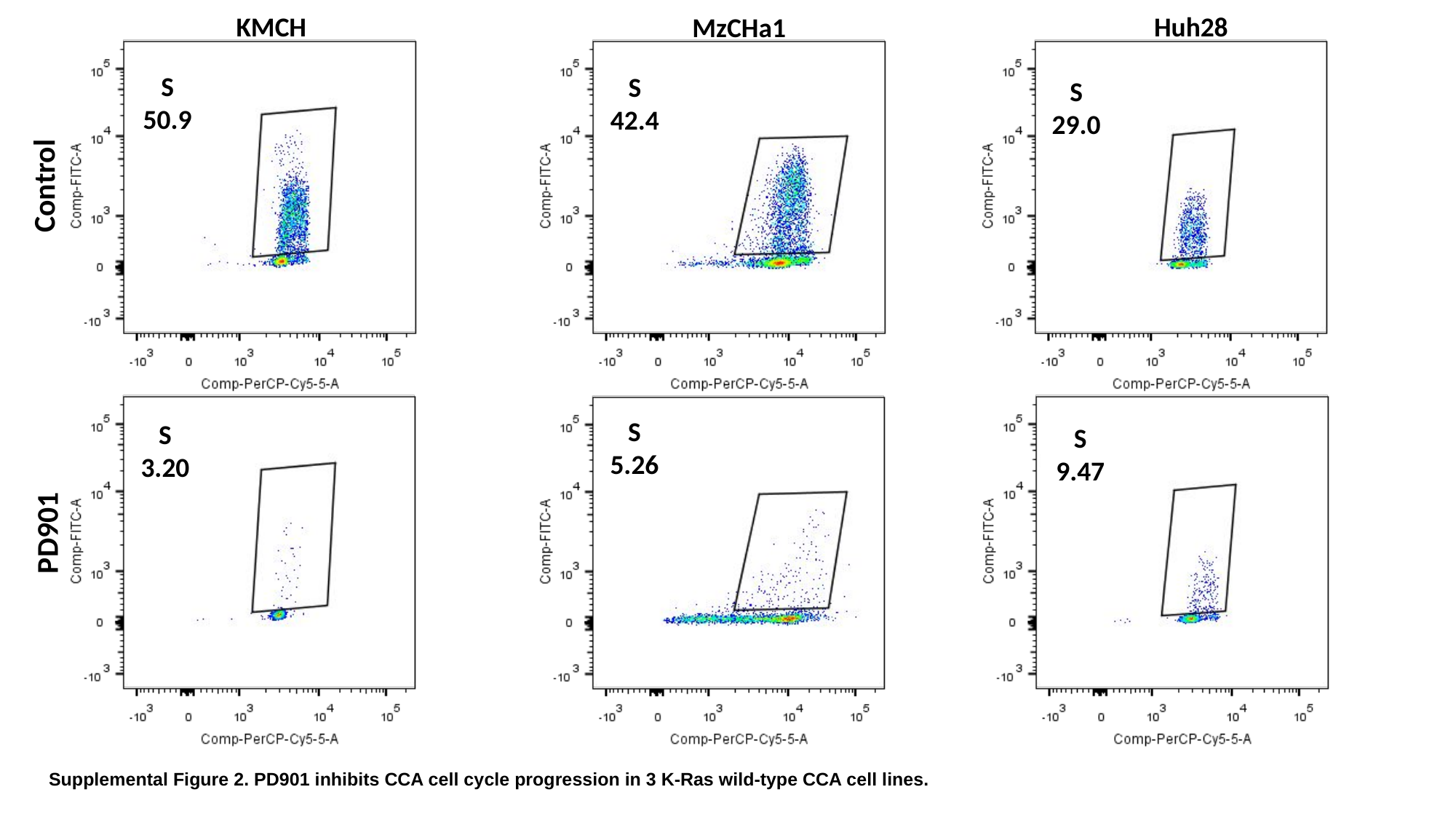

KMCH
Huh28
MzCHa1
S
50.9
S
42.4
S
29.0
Control
S
3.20
S
5.26
S
9.47
PD901
Supplemental Figure 2. PD901 inhibits CCA cell cycle progression in 3 K-Ras wild-type CCA cell lines.

## Slide 3
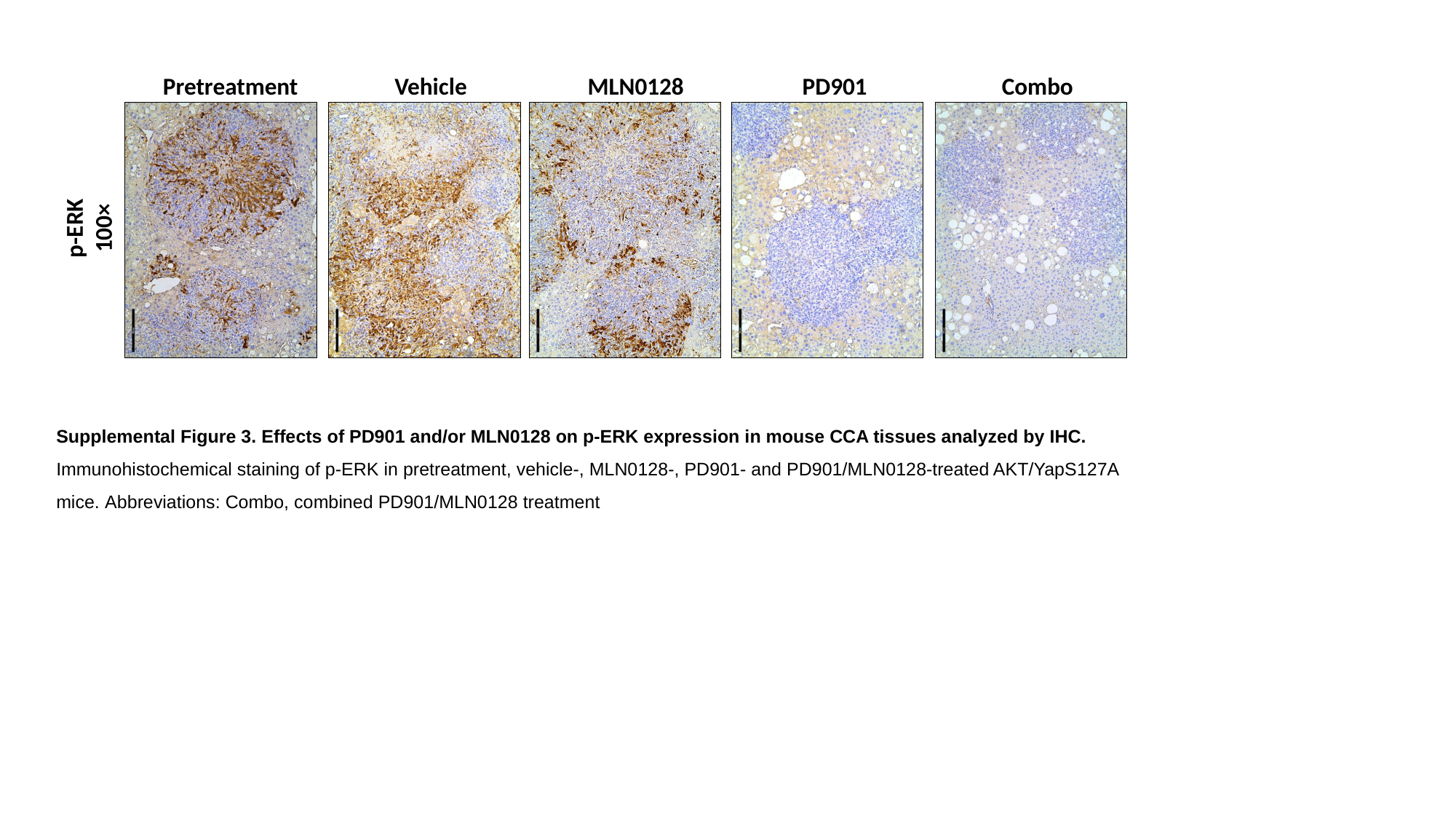

Pretreatment
Vehicle
MLN0128
PD901
Combo
p-ERK
100×
Supplemental Figure 3. Effects of PD901 and/or MLN0128 on p-ERK expression in mouse CCA tissues analyzed by IHC. Immunohistochemical staining of p-ERK in pretreatment, vehicle-, MLN0128-, PD901- and PD901/MLN0128-treated AKT/YapS127A mice. Abbreviations: Combo, combined PD901/MLN0128 treatment

## Slide 4
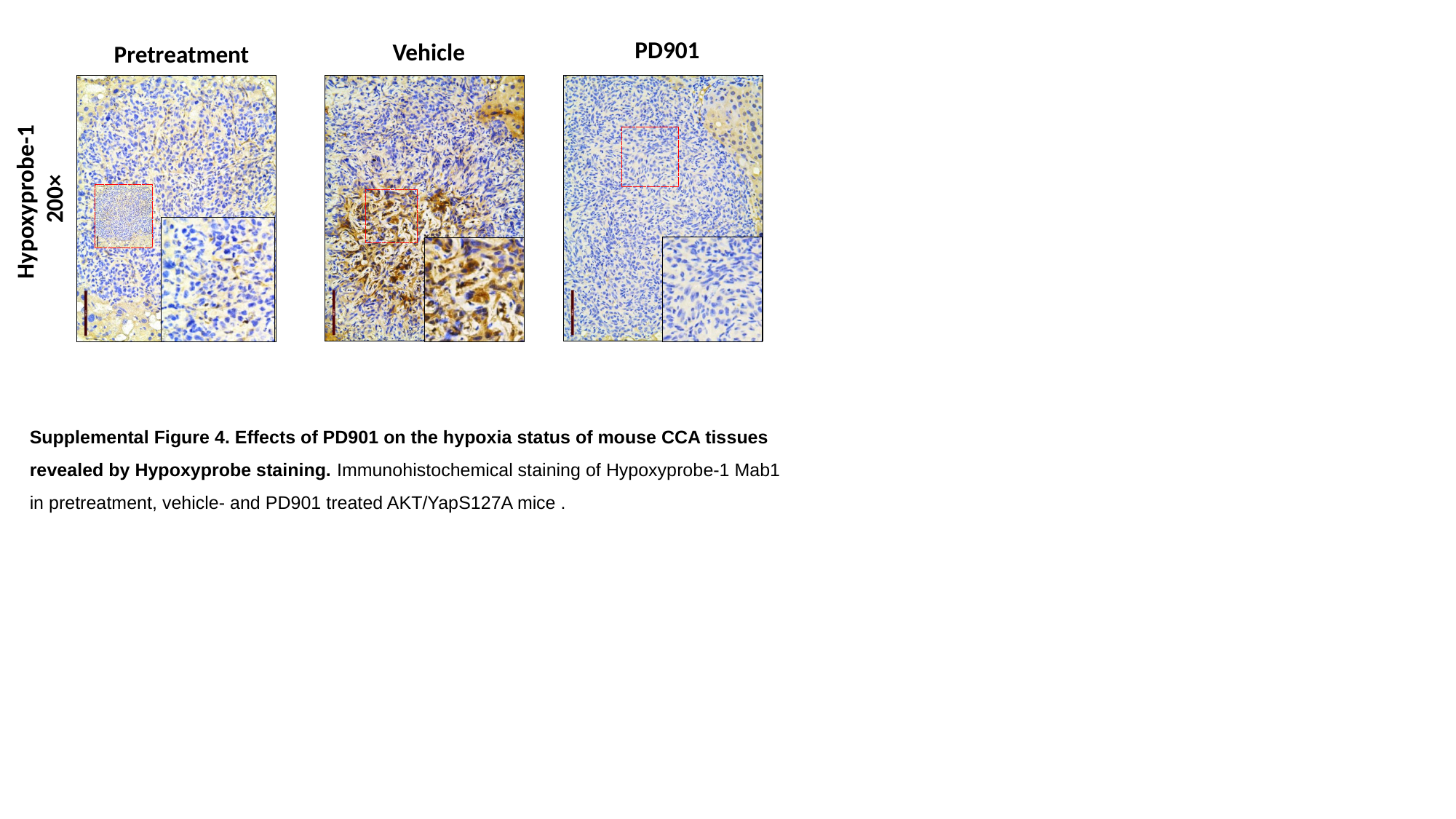

PD901
Vehicle
Pretreatment
Hypoxyprobe-1
200×
Supplemental Figure 4. Effects of PD901 on the hypoxia status of mouse CCA tissues revealed by Hypoxyprobe staining. Immunohistochemical staining of Hypoxyprobe-1 Mab1 in pretreatment, vehicle- and PD901 treated AKT/YapS127A mice .

## Slide 5
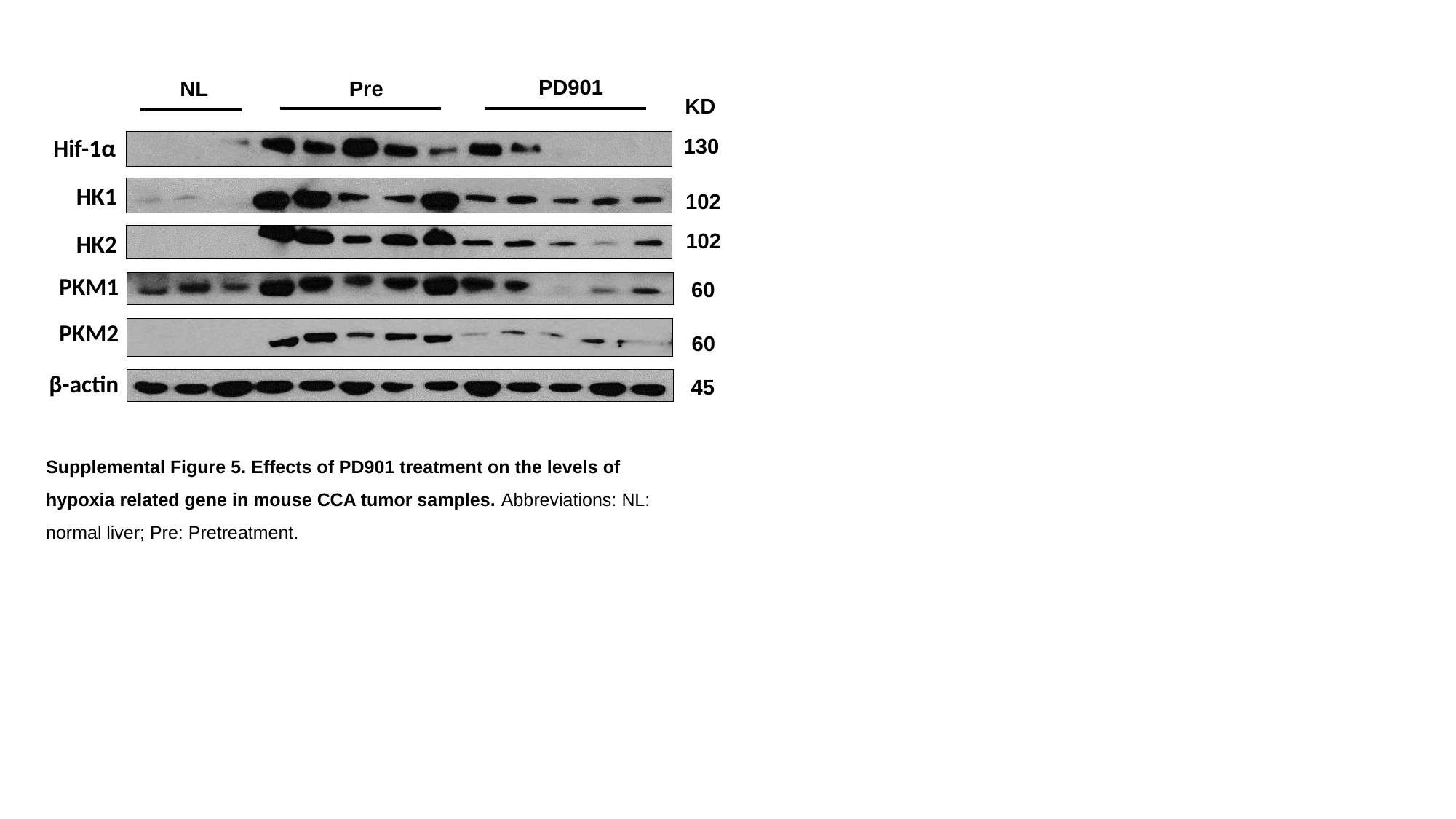

PD901
Pre
NL
KD
130
Hif-1α
HK1
102
102
HK2
PKM1
60
PKM2
60
β-actin
45
Supplemental Figure 5. Effects of PD901 treatment on the levels of hypoxia related gene in mouse CCA tumor samples. Abbreviations: NL: normal liver; Pre: Pretreatment.

## Slide 6
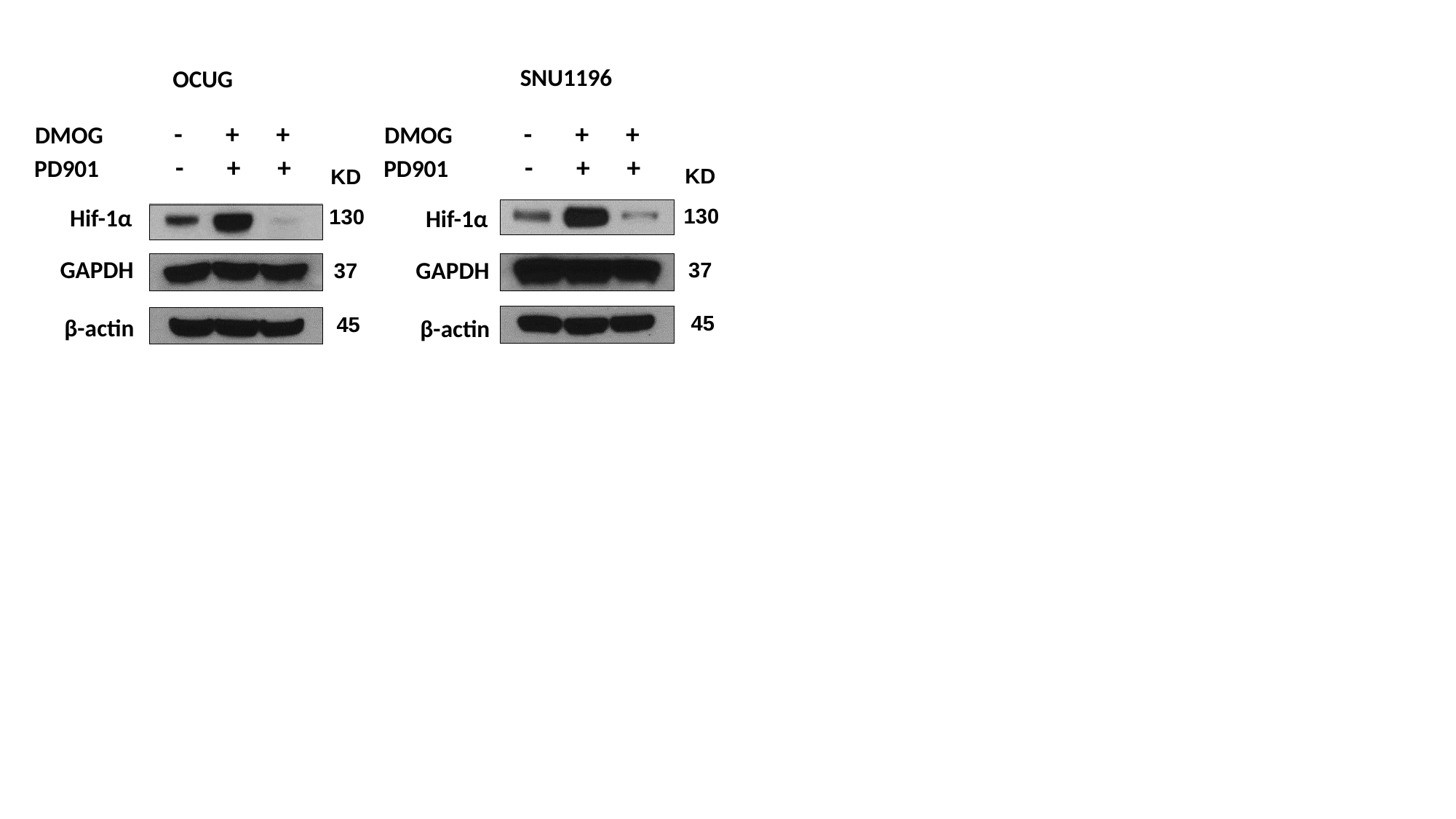

SNU1196
OCUG
DMOG - + +
PD901 - + +
DMOG - + +
PD901 - + +
KD
KD
130
Hif-1α
Hif-1α
130
GAPDH
GAPDH
37
37
45
45
β-actin
β-actin

## Slide 7
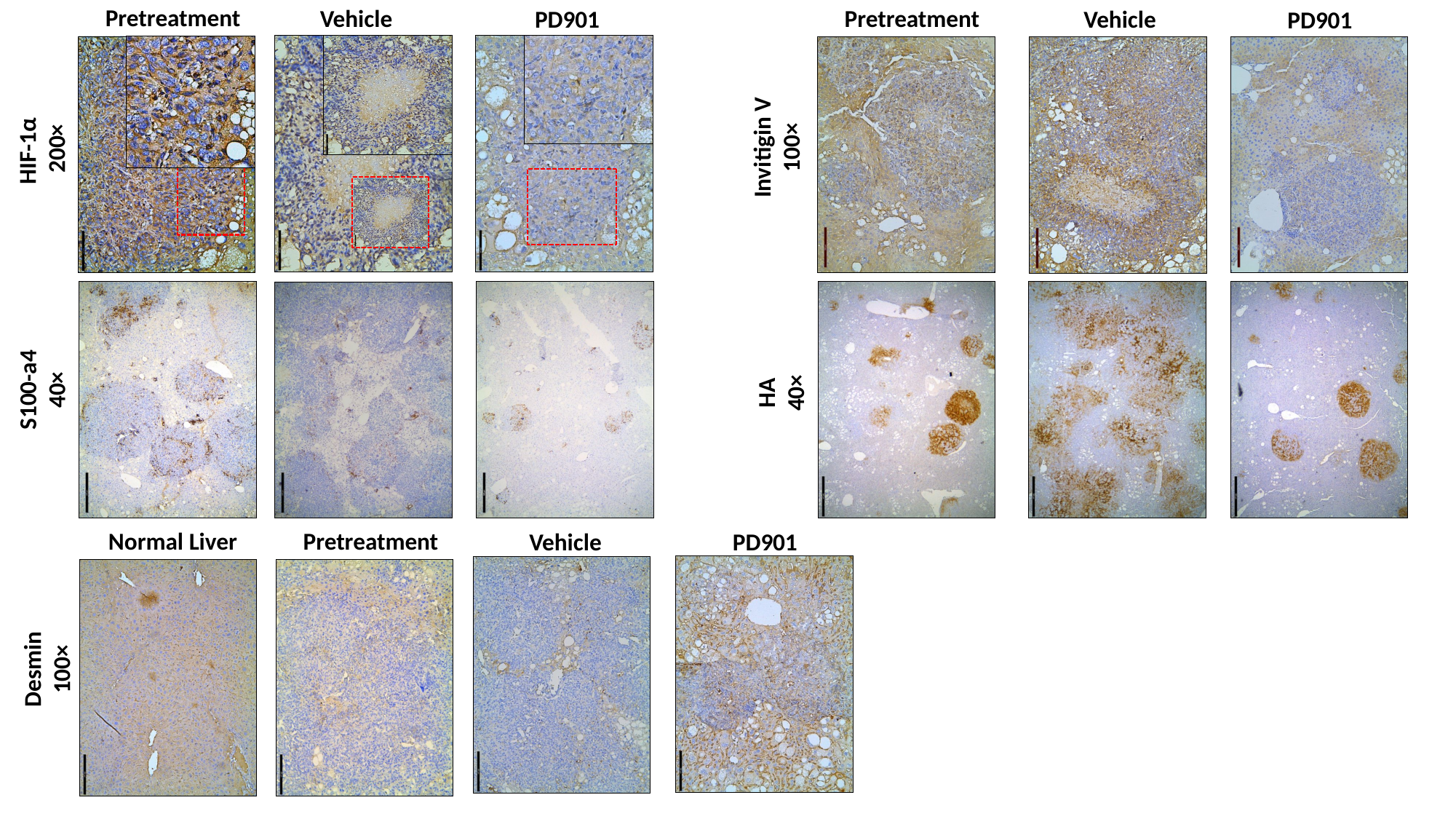

Pretreatment
Vehicle
Pretreatment
PD901
Vehicle
PD901
Invitigin V
100×
HIF-1α
200×
S100-a4
40×
HA
40×
Normal Liver
Pretreatment
Vehicle
PD901
Desmin
100×

## Slide 8
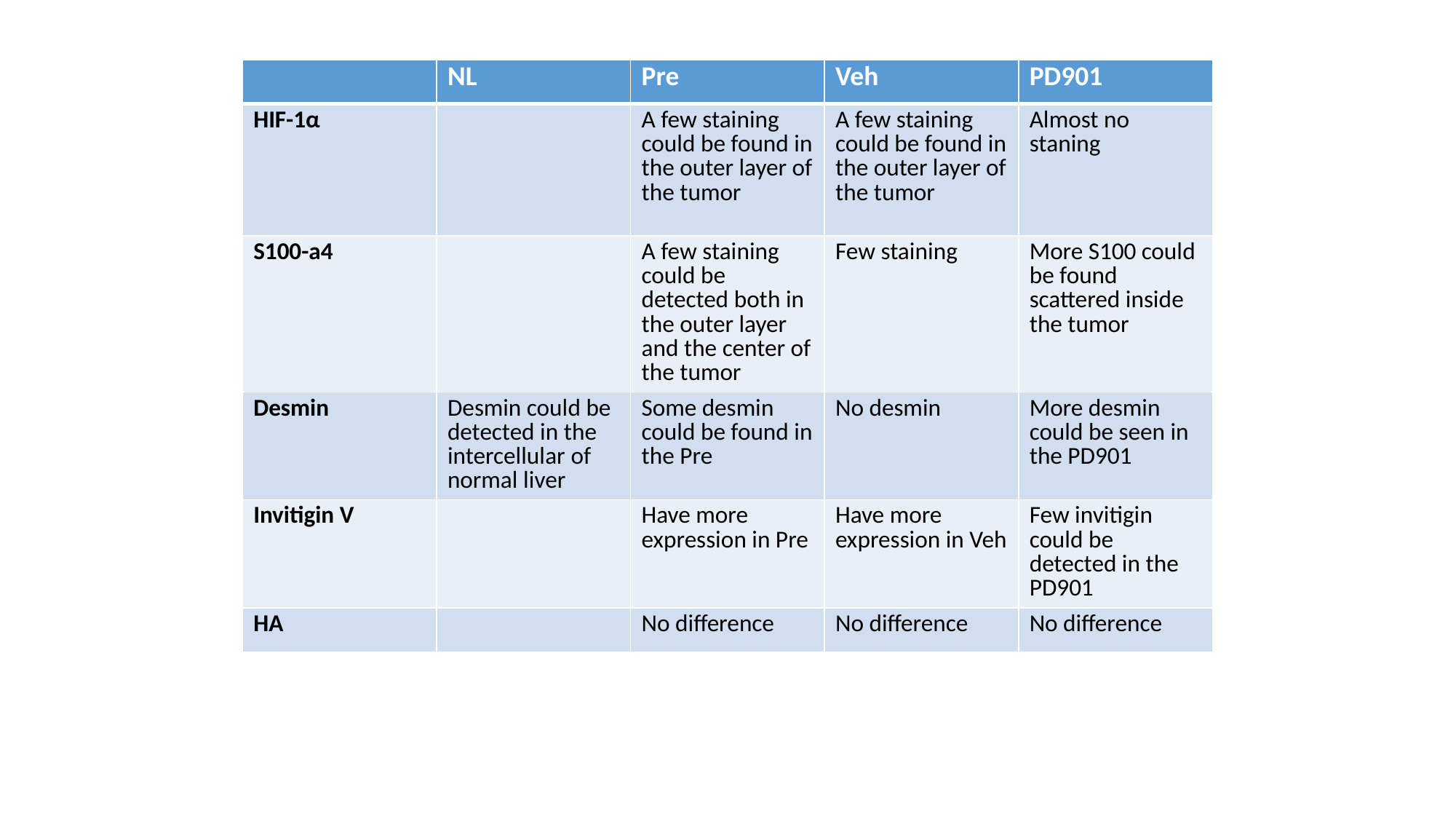

| | NL | Pre | Veh | PD901 |
| --- | --- | --- | --- | --- |
| HIF-1α | | A few staining could be found in the outer layer of the tumor | A few staining could be found in the outer layer of the tumor | Almost no staning |
| S100-a4 | | A few staining could be detected both in the outer layer and the center of the tumor | Few staining | More S100 could be found scattered inside the tumor |
| Desmin | Desmin could be detected in the intercellular of normal liver | Some desmin could be found in the Pre | No desmin | More desmin could be seen in the PD901 |
| Invitigin V | | Have more expression in Pre | Have more expression in Veh | Few invitigin could be detected in the PD901 |
| HA | | No difference | No difference | No difference |
